# Cross-cultural adaptation of the Mind-Wandering Questionnaire (MWQ) for Brazilian Portuguese and evidence of its validity

**DOI:** 10.47626/1516-4446-2023-3312

**Published:** 2024-03-06

**Authors:** Franciele Cristiane Peloso, Murilo Ricardo Zibetti, Antonio Egidio Nardi, Ramiro Figueiredo Catelan

**Affiliations:** 1 Programa de Pós-Graduação em Psicologia, Universidade do Vale dos Sinos, São Leopoldo, RS, Brazil; 2 Laboratório de Pânico e Respiração, Instituto de Psiquiatria (IPUB), Universidade Federal do Rio de Janeiro (UFRJ), Rio de Janeiro, RJ, Brazil; 3 Centro de Pesquisa em Devaneio Excessivo e Desregulação Emocional, IPUB, UFRJ, Rio de Janeiro, RJ, Brazil

**Keywords:** Attention, validation study, mind-wandering

## Abstract

**Objective:**

To adapt the Mind-Wandering Questionnaire (MWQ) for the Brazilian context and present evidence of validity based on its internal structure.

**Methods:**

A total of 2,682 Brazilian adults from different regions of the country took part in this study. Confirmatory factor analyses and multigroup confirmatory factor analyses (MGCFA) were performed to assess the factor structure of the MWQ. McDonald’s omega (ω) was generated to provide reliability indexes.

**Results:**

The analyses demonstrated an adequate factor structure for the MWQ adapted to the Brazilian context, corroborating the original article’s single-factor model and other adaptation studies. Furthermore, the results demonstrated the instrument’s reliability in a Brazilian population (ω = 0.88).

**Conclusion:**

The MWQ is thus an adequate, reliable, and quickly administered instrument for those whose aim is to measure deliberate and spontaneous MW in Brazil.

## Introduction

Mind-wandering (MW) refers to a shift from attentional focus on a primary task to automatic thoughts and feelings that are not related to the current task and/or are independent of external stimuli.^1^-^3^ It is important to highlight the differences between this phenomenon and other types of task-unrelated thoughts, such as daydreaming and rumination. Daydreaming can also refer to a stimulus-independent thought, but it does not necessarily involve a primary task. In contrast, MW presupposes a redirection of attention from the actual task to thoughts.^4^ Although rumination is also stimuli-independent and unrelated to the primary current task, it differs from MW because it encompasses rigid, automatic, and frequently negative thought content.^5^

The notion of MW involves a combination of executive control and a failure of executive control of attention, which reflects on the ability to control thoughts.^6^,^7^ This particularity leads to an understanding of MW as a deliberate or spontaneous process.^8^ Deliberate MW implies that an individual explicitly perceives their thoughts (metacognition) and intends to initiate or continue to engage in this process.^9^,^10^ On the other hand, in spontaneous MW, the individual is not consciously aware of the beginning of such episodes; thus, an automatic disconnection between perception and attention is produced.^1^,^11^ From this perspective, MW has been understood as a process opposite to mindfulness.^1^,^12^

MW can be perceived as a common phenomenon; it is estimated that humans spend 30 to 50% of their time wandering while awake on any given day.^13^,^14^ MW can serve as an adaptive function for individuals by providing benefits related to creative problem-solving, anticipation, planning of future goals (autobiographical planning), and boredom relief.^11^,^15^,^16^

Nonetheless, although recent studies have investigated the benefits of MW, others have documented disadvantages of the phenomenon. MW has been associated with cognitive disadvantages related to working memory, a decline in development of reading comprehension, reduced attention span on tasks, and poor executive control.^3^,^6^,^15^,^17^,^18^ Particularly in its spontaneous form, MW has been associated with negative mood and emotional dysregulation.^13^,^19^,^20^ Scientific studies suggest a correlation between attention deficit hyperactivity disorder (ADHD) and MW. There seems to be a correlation between severe ADHD and a higher frequency of MW, which may cause serious impairments of quality of life.^17^,^21^,^22^ Research has recently identified a correlation between higher stress levels and MW.^23^

The most common method for measuring MW involves interrupting individuals from time to time while they perform a task and verifying whether their attention is focused on the task or not.^2^ The Mind-Wandering Questionnaire (MWQ) was developed to measure the frequency of deliberate and spontaneous MW.^4^

The MWQ has been adapted and validated in other languages, including Spanish,^24^ Chinese,^25^ Croatian,^26^ Polish,^27^ and Japanese.^28^ It is one of the main instruments for assessing MW worldwide. All the studies mentioned above corroborated the results of the original study, showing adequate internal consistency and a single-factor instrument structure.

There is another instrument for assessing MW in Brazil, i.e., the Mind Excessively Wandering Scale (MEWS).^29^ However, the MEWS assesses excessive and/or pathological aspects of MW, whereas the MWQ conducts a more general assessment. The study of cross-cultural adaptation of the MEWS to the Brazilian context did not analyze the scale’s psychometric properties.^30^ Furthermore, the MWQ only has five items, whereas the MEWS comprises 12 items, meaning the latter is more suitable for Brazilian research applications. Considering such circumstances, the present study aims to adapt the MWQ to the Brazilian context and present evidence of validity based on its internal structure.

## Methods

### Procedures

This study is the outcome of a more extensive research project which aimed to investigate factors associated with maladaptive daydreaming, a form of pathological dissociation, in the Brazilian population. The following steps were taken to cross-culturally adapt the MWQ to Brazilian Portuguese:

1. Production of three translations conducted by three independent translators.
2. Synthesis of the translations.
3. Assessment of the synthesis by a committee of experts.
4. Test of the synthesis with the target population.
5. Back-translation by a native Anglophone.
6. Back-translation cross-check by the original author of the scale.
7. Production of the final version of the scale, considering the original author’s observations.
8. Data collection for validity evidence production.

First, three independent translators conducted three independent translations of the MWQ. Next, one of the researchers produced a synthesis of the translations to create a preliminary version of the translation. This version was then discussed by a committee formed by one of the researchers and four external consultants with broad clinical practice and considerable experience in development of psychometric instruments in order to assess the adequacy of the synthesis of the translations.

Thirty-nine participants were selected by invitation via social media networks to take part in the testing stage of the preliminary version of the instrument. They answered a questionnaire made available on the SurveyMonkey platform, which contained the five items of the MWQ followed by the following questions: 1) Is the language clear enough? 2) Is the language appropriate for your age group? 3) Did you understand the question? and 4) Does this item need to be modified (yes/no, with a blank space for participants to write their suggestions). Each item’s content validity index (CVI) was calculated after this data collection. All items were found to be suitable, with CVIs ranging from 0.918 to 0.954. Some minor suggestions made by participants were incorporated to increase the instrument’s clarity, appropriateness, and comprehensibility.

After this stage of the research, a version of the translation containing the participants’ feedback was sent for back-translation by a native Anglophone with experience in English grammar. This back-translation was then sent to one of the authors of the original MWQ for assessment. Based on his feedback, part of one of the items (item 5) was modified to maintain the original English meaning, and a final version of the MWQ in Brazilian Portuguese was produced. This version is available as Supplementary Material S1 (online-only).

This final version was made available together with the sociodemographic questionnaire and other instruments related to the main research project in an online survey in SurveyMonkey. The content was advertised on social media networks, particularly on Instagram, using paid traffic sources to reach more participants. We explicitly stated that only adults aged 18 years or older could participate. Social media advertisements asked participants if they tended to experience daydreaming or MW during their routine. By clicking on the advertisement, they were led directly to the SurveyMonkey link. Data collection was conducted between December 2021 and January 2022.

### Participants

A total of 2,682 individuals from all Brazilian regions participated in this study, mainly from the Southeast (47.2%), South (21.9%), and Northeast (16.3%) regions. They were aged from 18 to 69 years (M = 26.64; SD = 6.99), single (74.9%), with a maximum income of three times the Brazilian minimum monthly wage (73.6%), and had completed high school (48.7%) or an undergraduate degree (31.1%). The majority of the participants identified themselves as White (61.2%), followed by Mixed (27.2%), Black (9.5%), Asian (1.4%), and Indigenous (0.5%). Approximately half of the participants (n=217; 54.25%) reported having been diagnosed with a mental disorder. Table 1 shows the participants’ sociodemographic characteristics in the total sample and the two subsamples.

### Instruments

#### Sociodemographic profile

The participants informed their age, race/ethnicity, marital status, educational level, and income, among other variables.

#### Mind-Wandering Questionnaire (MWQ)^4^

The MWQ is a single-factor self-report scale composed of five items with a six-point Likert response scale, ranging from 1, almost never, to 6, almost always. Some of the items are as follows: “I have difficulty maintaining focus on simple or repetitive work,” “I do things without paying full attention,” and “I mind-wander during lectures or presentations.” The total MWQ score is obtained by summing its five items, which results in a minimum of 5 points and a maximum of 30 points. The original MWQ study showed evidence of its validity in adolescents and adults, reporting a Cronbach’s alpha of 0.85. The MWQ’s homogeneity was verified through factor analysis, which demonstrated that the five items explain 63.16% of the total variance of a single construct.^4^

#### Depression, Anxiety, and Stress Scale (DASS-21)^31^,^32^

The DASS-21 is a 21-item questionnaire that assesses symptoms of depression, anxiety, and stress. Each question is scored on a four-point scale, ranging from never to almost always. The questionnaire is divided into three subscales: depression (seven items), anxiety (seven items), and stress (seven items).

#### Adult ADHD Self-Report Scale (ASRS-18)^33^

The ASRS-18 is an 18-item screening tool designed to identify the potential presence of ADHD. This scale evaluates symptoms of inattention (nine items) and hyperactivity/impulsivity (nine items) by asking participants to indicate how frequently they have experienced these symptoms over the past 6 months. Participants rate each item on a five-point scale, ranging from never to very frequently.

#### Maladaptive Daydreaming Scale (MDS-16)^34^

The MDS-16 is a 16-item instrument designed to measure excessive daydreaming. It consists of three dimensions: craving, kinesthesia, and impairment. Participants rate the extent to which they identify with each statement on a scale from 0 to 100.

### Data analysis

Data from the answers of 2,682 participants were extracted from the data collection platform SurveyMonkey and imported into a database in SPSS version 23.0. First, a frequency analysis of “missing” MWQ items (> 0.1%) was performed. The missing data were considered missing completely at random (MCAR) and were thus replaced using the multiple imputation procedure. To maintain the possibility of data analysis by means of ordinal statistics, the imputed values were rounded to values compatible with the instrument’s original scale. A random subsample of 400 participants from this database was analyzed in this study. This subsample exceeds the recommended 10 participants per item for this genre of analysis, even considering the samples for invariance analysis.^35^

The analyses to accrue evidence of validity in relation to external variables were conducted using JASP (0.16.0.0) software. For this purpose, Pearson coefficients were calculated for the correlations between the MWQ scores and the MDS-16, DASS-21, and ASRS-18 scores.

The analyses to accrue evidence of validity according to its internal structure were conducted with a confirmatory factor analysis (CFA) performed using the same software and testing a single-factor model of the instrument’s structure. The invariance of the factor, metric, and scalar structure were tested according to the self-report presence of having received a psychiatric disorder diagnosis at any time in life, using a multigroup CFA (MGCFA) to evaluate the comparability of results in clinical vs. non-clinical groups, which could generate differences in the representation of the construct.

The CFA procedure was conducted using the robust method and both the nature of the items (ordinals) and deviations from normality were considered. Therefore, the robust diagonally weighted least squares (RDWLS) estimator (suitable for this data pattern) was used.^36^ The criteria used to evaluate the fit of the CFA model were taken from Brown,^37^ namely: chi-square (χ^2^) test not significant (p > 0.05); a ratio of χ^2^ to degrees of freedom (df) less than or equal to 3; comparative fit index (CFI) greater than 0.95; Tucker-Lewis index (TLI) greater than 0.95; standardized root mean residual (SRMR) less than or equal to 0.08; root mean square error of approximation (RMSEA) less than 0.06, and maximum 90%CI of 0.10.

Cheung & Rensvold’s^38^ criterion was used to interpret invariance in the MGCFA. This criterion suggests that changes greater than 0.01 in CFI indicate that the groups’ responses are not comparable (or invariant) (ΔCFI > 0.01). Once the factor structure had been established, internal consistency was evaluated using McDonald’s omega.

### Ethics statements

All the research procedures were reviewed and approved by the institutional ethics board at the Instituto de Psiquiatria, Universidade Federal do Rio de Janeiro (CAEE 49784521.1.0000.5263). The sample was recruited by convenience. Individuals who agreed to take part in the research signed an informed consent form, which defined their rights and described the risks and benefits of their participation. Anonymity was guaranteed, and the data were only accessed by the researchers, in accordance with the ethical considerations of the Declaration of Helsinki on research involving human beings.

## Results

The Pearson coefficients showed that the strongest correlations with the MWQ score were with the MDS-16 and the ASRS-18 inattention scale, which is commonly related to the MW construct. These data are shown in Table 2.

The CFA results for the single-factor model indicate model fit (χ^2^ = 9.999, df = 5; p = 0,075). Table 3 shows the fit indices for the model and factor loadings for the items.

Table 4 shows fit indices for the MGCFA.

The results demonstrate the scale’s invariance regardless of region, i.e., they indicate that use of the MWQ in different regions of Brazil is appropriate, despite the cultural differences related to the size of the country.

## Discussion

The present study aimed to adapt the MWQ to the Brazilian context and present evidence of validity based on its internal structure. This is a self-report instrument that evaluates the frequency of both deliberate and spontaneous MW. With the addition of a few adaptations suggested by Borsa et al.,^39^ this study adopted the guidelines from the International Test Commission (ITC)^40^ for adaptation of the model and acquisition of evidence of its validity.

The factor structure of the MWQ is stable as a single-factor measurement in different cultures.^24^-^28^ When analyzed with a CFA, the factor model of the MWQ maintained its single-factor structure in Brazil. The results indicate an adequate fit to the data, as reported in other parts of the world. In comparison to the original study^4^ and to other adaptations, the MWQ exhibited a stable and reliable structure for Brazilian adults.

The scale has demonstrated high internal consistency and homogeneity in the Brazilian sample. The values obtained for scale reliability using McDonald’s Omega (ω = 0.88) were adequate for all items and similar to those found for the original version and in other adaptation studies of the instrument. In order to verify the invariance of the factor, metric, and scalar structure, an MGCFA was performed for those with a history of a psychiatric diagnosis vs. those who had never had such a diagnosis. The results demonstrated measurement invariance, which shows that it is possible to compare MWQ scores obtained from clinical and non-clinical groups.

The MWQ is a reliable instrument for those who aim to measure both deliberate and spontaneous MW, rather than excessive daydreaming or rumination, for example. The MWQ is a measurement tool that evaluates the frequency of MW without focusing on excessive or pathological aspects, unlike other instruments already adapted for the Brazilian population. The MWQ is also a short measure (five items), quick to administer, and easily understood by different populations due to its simple language. Therefore, the moderate correlation with MDS-16 also demonstrates evidence of validity in relation to this external variable, since they have many similarities, although they are not the same phenomenon.

MW has been associated with different clinical conditions, such as learning difficulties, ADHD, increased stress levels, low self-esteem, and mood swings.^17^-^23^ Therefore, the significant correlations with the ASRS-18 and the DASS-21 provide evidence of validity based on external variables. The greater strength of the correlation with inattention (ASRS-18) than with the correlations observed with impulsivity (ASRS-18), stress, depression, and anxiety (DASS-21) provides further support for external validity since inattention is a closer construct to MW than the others. Future studies might investigate the correlation between MWQ scores and positive outcomes, since MW has also been identified as a creative phenomenon contributing to problem-solving and boredom relief.^11^,^16^

This study adapting the MWQ to the Brazilian context and collecting evidence of its validity has important limitations. One concerns the lack of instruments to verify the MWQ’s validity based on its relations with external measures. Additionally, the sample is mainly composed of women, which may indicate potential bias. It is important to highlight that one of the limitations related to use of this instrument concerns item 5. Those who are not exposed to academic activities such as lectures and classes (e.g., people with simpler life functions) may not respond adequately to this item. This, however, does not compromise the face validity of the instrument, which might be more suitable for samples with higher education. Further studies are necessary to accumulate additional evidence to endorse interpretations of the MWQ scores for the Brazilian population.

This study has produced a first version of the MWQ for the adult Brazilian population, with adequate reliability and internal scale validity. The study corroborates the data found in the original article and by other adaptation studies worldwide, finding a single-factor internal structure for the MWQ. These findings constitute initial evidence of validity for this instrument in Brazil. The MWQ could be useful in different contexts; its aim is to measure the frequency of MW, which is a common psychological phenomenon in the general population and in many clinical conditions.

Our results were obtained from a sample composed mainly of Brazilian people who declared themselves to be women (78%), young (mean age 26 years), white (61%), living in the South and Southeast regions (68%), and educated to at least secondary level (98%). Thus, for use with the very diverse broader Brazilian population, another study would be necessary. Administration of the MWQ in a face-to-face interview or using self-report formats (both common in Brazilian health services) may also yield different results from those obtained in online surveys.

## Data availability

Original data from this study are available upon request to the corresponding author.

## Disclosure

The authors report no conflicts of interest.

## Figures and Tables

**Table 1:** Sociodemographic characteristics

Characteristic	Total sample (n=2,682)
Age, median (SD)	26.64 (6.99)
Geographic region	
Southeast	1,266 (47.20)
South	588 (21.92)
Northeast	437 (16.29)
Mid-West	245 (9.13)
North	129 (4.81)
Not reported	17 (0.63)
Marital status	
Single	2,009 (74.91)
Civil union	309 (11.52)
Married	297 (11.07)
Divorced	63 (2.35)
Not reported	4 (0.15)
Income† (multiples of minimum wage)	
No income	788 (28.38)
< 1	602 (22.45)
1-3	611 (22.78)
3-6	485 (18.08)
6-9	103 (3.84)
9-12	41 (1.53)
12-15	22 (0.82)
> 15	21 (0.78)
Not reported	9 (0.34)
Highest educational level completed	
None	5 (0.19)
Elementary school	56 (2.09)
High school	1,306 (48.69)
Undergraduate degree	835 (31.13)
MBA or specialization	318 (11.86)
Master’s degree	130 (4.85)
PhD degree	29 (1.08)
Not reported	3 (0.11)
Gender	
Cisgender women	2,092 (78.00)
Cisgender men	430 (16.00)
Other‡	153 (5.74)
Not reported	7 (0.26)
Race/ethnicity	
White	1,641 (61.18)
Mixed	729 (27.18)
Black	255 (9.51)
Asian	38 (1.42)
Indigenous	14 (0.52)
Not reported	5 (0.18)

Data presented as n (%), unless otherwise specified.

MBA = Master of Business Administration degree.

† At the time of this study, the minimum wage in Brazil equated to approximately 232 USD per month.

‡ Including transgender men, transgender women, and non-binary individuals.

**Table 2:** Pearson’s coefficients for correlations between MWQ and external variables

	1.0	2.0	3.1	3.2	4.1	4.2	4.3
1. MWQ	-						
2. MDS	0.516	-					
3. ASRS-18							
3.1 Attention	0.729	0.541	-				
3.2 Impulsivity	0.416	0.378	0.480	-			
4. DASS-21							
4.1 Depression	0.319	0.437	0.353	0.254	-		
4.2 Anxiety	0.250	0.427	0.313	0.350	0.623	-	
4.3 Stress	0.338	0.448	0.416	0.506	0.682	0.739	-
4.4 Total	0.343	0.493	0.407	0.410	0.883	0.876	0.902

All correlations were significant to p < 0.01.

ASRS-18 = Adult ADHD Self-Report Scale; DASS-21 = Depression, Anxiety, and Stress Scale; MDS = Maladaptive Daydreaming Scale; MWQ = Mind-Wandering Questionnaire.

**Table 3:** CFA of the MWQ using the DWLS estimator

MWS items	Factor loadings (95%CI)	Residual covariance
1. I have difficulty maintaining focus on simple or repetitive work.	0.757 (0.704-0.809)	0.427
2. While reading, I find I haven't been thinking about the text and must therefore read it again.	0.773 (0.728-0.818)	0.403
3. I do things without paying full attention.	0.842 (0.805-0.880)	0.290
4. I find myself listening with one ear, thinking about something else at the same time.	0.825 (0.786-0.863)	0.320
5. I mind-wander during lectures or presentations.	0.817 (0.778-0.857)	0.332
Fit indices		
χ^2^(df); p		9.999(5); p = 0.075
CFI		0.999
TLI		0.998
SRMR		0.025
RMSEA		0.050 (0.000-0.095)
Reliability		
McDonald’s omega 95%CI (LL-UL)		0.880 (0.862-0.899)

CFA = confirmatory factor analysis; CFI = comparative fit index; df = degrees of freedom; DWLS = diagonally weighted least squares; p = test statistics; RMSEA = root mean square error of approximation; SRMR = standardized root mean square residual; LL = lower limit; UL = upper limit; TLI = Tucker-Lewis index; χ^2^ = chi-square.

**Table 4:** Fit indices for the MGCFA

Measurement invariance	Fit indices				
MWQ	RMSEA (90%CI)	SRMR	TLI	CFI	DCFI
Southeast vs. other regions					
Configural invariance	0.048 (0.000-0.098)	0.031	0.998	0.999	-
Metric invariance	0.060 (0.009-0.0100)	0.040	0.997	0.998	-0.001
Scalar invariance	0.007 (0.000-0.053)	0.034	1.000	1.000	+0.002

CFI = comparative fit index; DCFI = CFI chi-square; MGCFA = multigroup confirmatory factor analyses; SRMR = standardized root mean square residual; TLI = Tucker-Lewis index.
