# Supplementary material for: Cross-cultural adaptation of the Mind-Wandering Questionnaire (MWQ) for Brazilian Portuguese and evidence of its validity
Source: Braz J Psychiatry. 2024 Mar 6;46:e20233312. doi: 10.47626/1516-4446-2023-3312 (PMC11189109; doi:10.47626/1516-4446-2023-3312)
Supplement: Supplementary file 1 [file bjp-46-e20233312-s001.pdf]

http://doi.org/10.47626/1516-4446-2023-3312

Supplementary Material S1

Questionário de Divagação Mental

Mind-Wandering Questionnaire (MWQ)

VERSÃO ORIGINAL: Michael D. Mrazek et al. (2013)†
TRADUÇÃO E ADAPTAÇÃO: Franciele Cristiane Peloso, Murilo Ricardo Zibetti, Antonio Egidio Nardi e Ramiro Figueiredo Catelan

---

Por favor, siga as instruções abaixo para responder ao questionário:

A seguir encontra-se uma sequência de frases sobre suas experiências do dia a dia. Usando a escala de 1 a 6 abaixo, por favor indique o quão frequentemente ou infrequentemente você tem cada uma dessas experiências.Por favor, responda de acordo com o que **realmente acontece na sua experiência** em vez de responder sobre como você acha que sua experiência deveria ser. Leia atentamente cada item e **marque a resposta que melhor corresponde à sua experiência.**

		Quase nunca	Muito raramente	Às vezes	Frequentemente	Muito frequentemente	Quase sempre
1	Tenho dificuldade em manter o foco em atividades simples ou repetitivas.	1	2	3	4	5	6
2	Enquanto estou lendo um texto, percebo quenão estou prestando atenção nele, então preciso lê-lo de novo.	1	2	3	4	5	6
3	Eu faço as coisas sem prestar totalmenteatenção nelas.	1	2	3	4	5	6
4	Me pego ouvindo as coisas pela metade, pensando em outra coisa ao mesmo tempo.	1	2	3	4	5	6
5	Meus pensamentos vão para outro lugar durante aulas, palestras ou apresentações.	1	2	3	4	5	6

† Mrazek MD, Phillips DT, Franklin MS, Broadway JM, Schooler JW. Young and restless: validation of the Mind-Wandering Questionnaire (MWQ) reveals disruptive impact of mind-wandering for youth. Front Psychol. 2013;4:560.
